# Clinicopathologic characteristics of second primary squamous cell carcinoma in patients with nasopharyngeal carcinoma after intensity-modulated radiotherapy

**DOI:** 10.1038/s41598-023-34848-8

**Published:** 2023-05-20

**Authors:** Xi Wang, Shunlan Wang, Yang Cao, Chunqiao Li, Caishan Fang, Weiping He, Zhuming Guo

**Affiliations:** 1 Department of Otolaryngology, Guangzhou University of Traditional Chinese Medicine First Affiliated Hospital, Guangzhou, China; 2 The First School Of Clinical Medicine, Guangzhou University of Chinese Medicine, Guangzhou, China; 3 Department of Oncology, Guangzhou University of Traditional Chinese Medicine First Affiliated Hospital, Guangzhou, China; 4 Department of Head and Neck, Sun Yat-Sen University Cancer Center, Guangzhou, China

**Keywords:** Cancer, Oncology, Risk factors

## Abstract

To compare the clinicopathologic characteristics of second primary squamous cell carcinoma (SPSCC) in patients with nasopharyngeal carcinoma (NPC) after intensity-modulated radiotherapy (IMRT) with that after radiotherapy (RT). From 49,021 patients with NPC who treated by definitive RT, we were able to identify 15 male patients with SPSCC after IMRT, and 23 male patients with SPSCC after RT. We examined the difference between groups. In IMRT group, 50.33% developed SPSCC within 3 years, whereas 56.52% developed SPSCC after more than 10 years in RT group. Receiving IMRT was related positively to an increased risk of SPSCC (HR = 4.25; *P* < 0.001). There was no significant correlation between receiving IMRT and the survival of SPSCC (*P* = 0.051). Receiving IMRT was related positively to an increased risk of SPSCC, and the latency was much shorter. A follow-up protocol, especially in the first three years, should be designed for NPC patients with IMRT.

## Introduction

Exposure to ionizing radiation is a known risk factor for cancer and is related to the development of second primary carcinoma (SPC)^1^. The increasing incidences of SPC attributable to radiotherapy (RT) have become a major concern among long-term survivors of nasopharyngeal carcinoma (NPC)^2^. Although intensity-modulated radiotherapy (IMRT) provided impressive local control in the treatment of primary NPC^3,4^, the use of more fields led to an increase in the total volume that received radiation (mostly low-dose). It is known that there is a dose–response relation between SPC and radiation at low doses, but not at high doses^1^.

We found that the median latency halved in patients diagnosed of NPC after 2000 when we began to use IMRT in the treatment of NPC^5^. The prevailing use of IMRT in the treatment of NPC may have a substantial effect on the incidence of SPC. The authors sought to learn if the clinicopathologic characteristics of second primary squamous cell carcinoma (SPSCC) in patients with nasopharyngeal carcinoma (NPC) after IMRT is similar to that observed after RT. To investigate this aspect, we have therefore performed a retrospective analysis the clinical and survival data of 15 patients with SPSCC after IMRT of NPC and 23 patients with SPSCC after RT of NPC.

## Materials and methods

The Sun Yat-Sen University Cancer Center is the largest therapy and diagnosis center of NPC in southeastern China. According to the institutional electronic medical records, a total of 49,021 patients with NPC were treated by definitive RT in our Cancer Center between January 1970 and December 2009 and information about their pretreatment characteristics, therapy, and outcome is available. From this database we were able to identify 15 male patients for IMRT group who (1) had second primary squamous cell carcinoma (SPSCC) arising in the head and neck, (2) were treated only by IMRT for NPC, (3) had no smoking and excessive alcohol intake history, and (4) had no family history of cancer. We were able to identify 23 male patients for RT group who (1) had SPSCC arising in the head and neck, (2) were treated only by RT for NPC, (3) had no smoking and excessive alcohol intake history, and (4) had no family history of cancer. Prior informed consent was obtained from patients, as was the approval of the Institutional Research Ethics Committee of the Sun Yat-sen University Cancer Center.

All patients in IMRT group were immobilized with a customized thermoplastic head–neck–shoulder cast in the supine treatment position. Two sets of images with and without contrast were obtained from the computed tomography (CT) simulator for treatment planning. The definition of target volumes was in accordance with the International Commission on Radiation Units and Measurements (ICRU) report 50 and 62^6,7^. The gross tumor volume (GTV) is defined as the whole known gross extent of the primary nasopharyngeal tumor and involved lymph nodes determined from magnetic resonance imaging (MRI), physical examination and endoscopy. The first clinical tumour volume (CTV1) was defined as the GTV plus a margin of 5–10 mm for potential microscopic spread, including the entire nasopharyngeal mucosa plus a 5-mm submucosal volume. The second CTV (CTV2) was defined by adding a margin of 5–10 mm to CTV1 and included the following regions, which required prophylactic irradiation: the retropharyngeal lymphnode regions, clivus, skull base, pterygoid fossae, parapharyngeal space, inferior sphenoid sinus, posterior edge of the nasal cavity, maxillary sinuses, and lymphatic drainage area. The planning target volume (PTV) for GTV and CTVs were generated automatically by adding a 5-mm margin after delineation of tumour targets according to the immobilization and localization uncertainties. The prescribed dose was 60–80 Gy (mean ± SD, 70.40 ± 4.48 Gy) to the PTV of the GTV, 54–66 Gy to the PTV of the CTV1 (PTV1), and 50–56 Gy to the PTV of the CTV2 (PTV2) in 28–35 daily fractions, respectively. The doses limited to the major organs at risk (OAR) were as follows: brainstem: Dmax < 54 Gy; spinal cord: Dmax < 45 Gy; optic nerve and chiasm: Dmax < 50 Gy; temporal lobes: Dmax < 60 Gy; parotid glands: Dmean < 26 Gy; mandible: Dmax < 70 Gy; oral cavity: Dmax < 40 Gy; glottis: Dmean < 45 Gy; and cervical esophagus: Dmean < 45 Gy.

All patients in RT group were immobilized in the supine position with a thermoplastic mask and treated with two lateral opposing faciocervical portals to irradiate the nasopharynx and upper neck in one volume followed by application of the shrinking-field technique to limit irradiation of the spinal cord. An anterior cervical field was used to treat the neck with a laryngeal block. The accumulated radiation doses were 59–80 Gy (mean ± SD, 68.09 ± 6.20 Gy), with 2 Gy per fraction applied to the primary tumour and 50–55 Gy applied to the uninvolved areas.

Criteria set by Warren and Gates^8^ were used to define the SPC. All tumors were confirmed pathologically as distinct malignancies. None were undifferentiated carcinomas, excluding the possibility of locoregional recurrence or distant metastasis of nasopharyngeal origin. For the distinction between a recurrence, metastasis, or SPC, we used case-by-case judgment instead of rigid definitions. All patients were staged according to the 2002 American Joint Committee on Cancer (AJCC) staging system. TNM classification was based on pathological information; clinical information was used if pathology data were missing.

Follow-up data were collected from the outpatient service and complementary data were obtained by telephone inquiry and follow-up letters. They were reviewed every month for the first year postoperatively, every two months during the second year, 4-monthly during the third and fourth years, and 6-monthly thereafter. The cutoff date of the last follow-up was December 31, 2014 for the censored data analysis. Follow-up time was calculated from the time of diagnosis of SPSCC to the last date of contact. At the time of data collection, all of the patients had been followed for a minimum of 5 years after therapy. The median follow-up period for all patients was 61 months (range, 6–288 months).

The ANOVA test were used to examine the difference between groups. Overall survival (OS) was calculated from the diagnosis of SPSCC until death or last follow-up. The Cox proportional hazard model was used for analysis of estimated cumulative risk and OS of all SPSCC from the date of initial treatment. The statistical analysis was performed using SPSS 25.0 software (SPSS Inc., Chicago, IL). The difference was considered statistically significant when the *P* value was less than 0.05.

### Ethical approval

We confirm that all methods were performed in accordance with the relevant guidelines and regulation. The Guangzhou University of Traditional Chinese Medicine First Affiliated Hospital had approved the research, the approval letter number is JY2022-034. Informed consent was obtained from all patients.

## Results

The age at NPC diagnosis of IMRT group ranged from 37 to 65 years (mean ± SD, 52.67 ± 9.54 years), and the age at NPC diagnosis of RT group ranged from 30 to 59 years (mean ± SD, 42.78 ± 9.54 years). The difference in the age at NPC diagnosis between the two groups was not statistically significant (*P* = 0.945). The radiotherapy dose of IMRT group ranged from 60 to 80 Gy (mean ± SD, 70.40 ± 4.48 Gy), and the radiotherapy dose of RT group ranged from 59 to 80 Gy (mean ± SD, 68.09 ± 6.20 Gy). The difference in the radiotherapy dose between the two groups was not statistically significant (*P* = 0.068).

**Figure 1:**
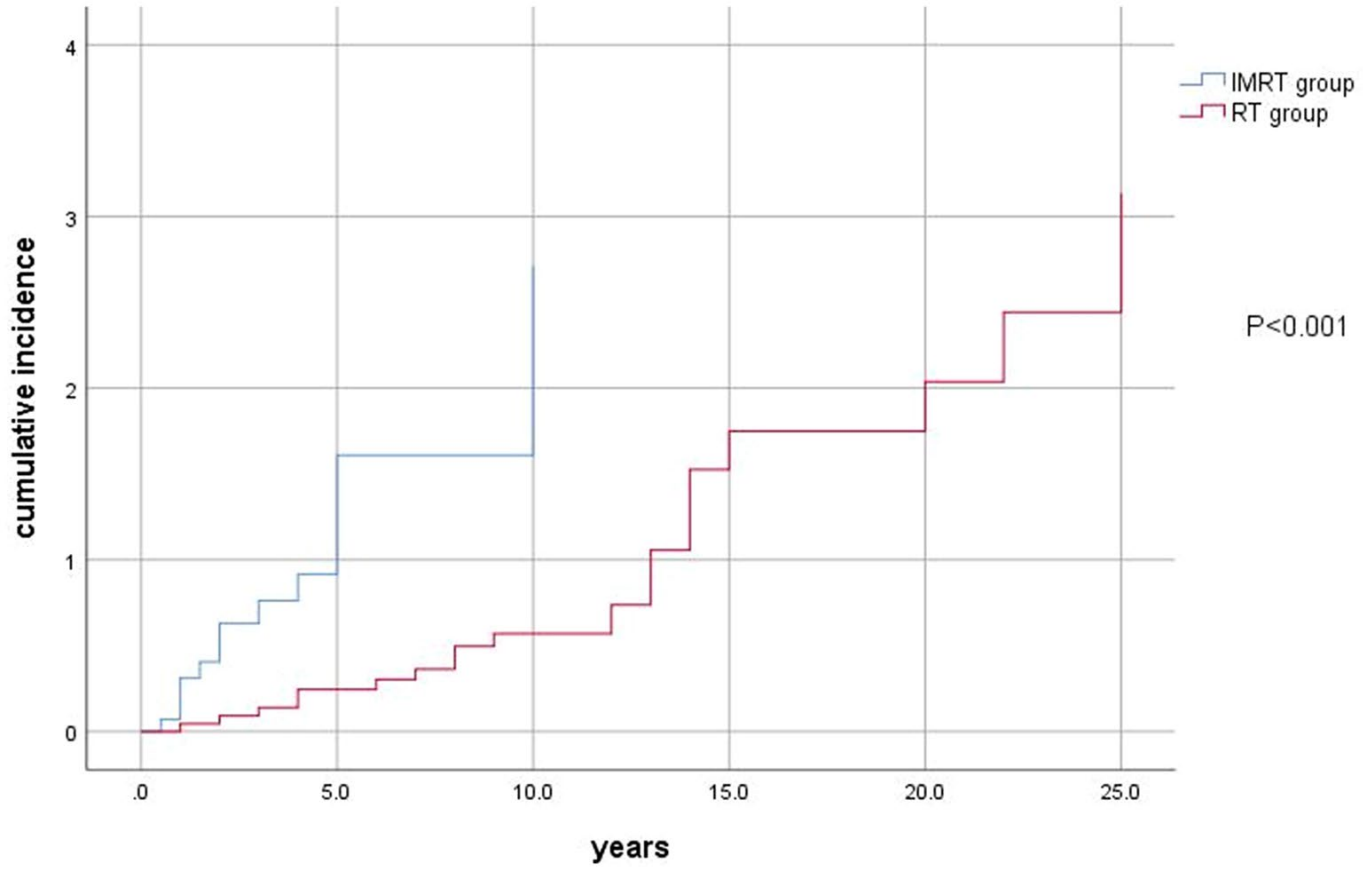
Cumulative incidence according to IMRT of SPSCC.

The main characteristics of the patients at diagnosis of SPSCC are summarized in Table 1. The age at SPSCC diagnosis of IMRT group ranged from 40 to 68 years (mean ± SD, 54.07 ± 9.31 years), and the age at SPSCC diagnosis of RT group ranged from 40 to 70 years (mean ± SD, 54.43 ± 7.70 years). The difference in the age at NPC diagnosis between the two groups was not statistically significant (*P* = 0.180). The latency of IMRT group ranged from 0.5 to 12 years (mean ± SD, 4.20 ± 3.71 years), and the latency of RT group ranged from 1 to 32 years (mean ± SD, 11.78 ± 7.65 years). Univariate analysis revealed that receiving IMRT was related positively to an increased risk of SPSCC (hazard risk [HR] 4.25; 95% CI 1.92–9.40; *P* < 0.001, Fig. 1). Eight (50.33%) patients developed SPSCC within 3 years in IMRT group, whereas 13 (56.52%) patients developed SPSCC after more than 10 years in RT group. The most common tumor site in the SPSCC of two groups was the oral cavity. The overall 3 and 5-year survival rates of the IMRT group were 40% and 26.67%. The overall 3 and 5-year survival rates of the RT group were 56.52% and 34.78%. Univariate analysis revealed that there was no significant correlation between receiving IMRT and the survival of SPSCC (*P* = 0.051).

**Table 1:** Characteristics of patients and tumors at the time of second primary squamous cell carcinoma diagnosis (*n* = 38).

Characteristic	IMRT group (*n* = 15)		RT group (*n* = 23)	
	No. of patients	%	No. of patients	%
Latency				
Within 3 years	8	53.33	3	13.04
Within 3–5 years	4	26.67	2	8.07
Within 5–10 years	2	13.33	5	21.74
More than 10 years	1	6.67	13	56.52
Location of disease				
Oral cavity				
Tongue	2	13.33	9	39.13
Gingiva	6	40	4	17.39
Hard palate	1	6.67	5	21.74
Bucca cavioris	3	20	1	4.35
Other sites				
Paranasal sinuses	0	0	1	4.35
Hypopharynx	0	0	1	4.35
Skin	1	6.67	1	4.35
Cervical esophagus	2	13.33	1	4.35
Histologic type				
Well-differentiated SCC	9	60	11	47.83
Moderately-differentiated SCC	3	20	7	30.43
Poorly-differentiated SCC	3	20	5	21.74
TNM stage				
I	4	26.67	7	30.43
II	4	26.67	7	30.43
III	6	40	8	34.78
IV	1	6.67	1	4.35
Treatment				
Surgery ± adjuvant RT	10	66.67	13	56.52
RT	2	13.33	2	8.07
Chemotherapy	1	6.67	4	17.39
Without treatment	2	13.33	4	17.39
Overall survival				
3-year OS	6	40	13	56.52
5-year OS	4	26.67	8	34.78

*IMRT* intensity-modulated radiotherapy, *RT* radiotherapy, *SCC* squamous cell carcinoma, *OS* overall survival.

## Discussion

Nasopharyngeal carcinoma is a radiosensitive tumor, excellent disease control can be achieved after definitive chemo-irradiation using IMRT technique. Since NPC is usually diagnosed at relatively young ages and most patients now survive with less advanced disease for a long time after RT^9,10^. However, such techniques may lead to long-term complications, including SPC, which have become increasingly important^9^. The increasing incidences of SPC attributable to RT have become a major concern among long-term survivors of NPC, and the prevailing use of IMRT in the treatment of NPC may have a substantial effect on the incidence of SPC^5^. In light of this, the authors sought to learn the clinicopathological characteristics, survival rate and potential risk of SPSCC in patients who received IMRT for NPC.

Compared with conventional radiotherapy, IMRT, with multifield rotational radiation, can increase the exposure of normal tissue or surrounding organs-at-risk to doses of radiation^11^. First, the increased size of the radiation field of IMRT can increase the lower doses exposure of a bigger volume of normal tissue^1,12,13^. It is estimated that an additional 0.5% of surviving patients will develop a second malignancy as a result of this factor^1^. Second, delivery of a specified dose to the isocenter from a modulated field, delivered by IMRT, will require the accelerator to be energized for longer (hence more monitor units are needed) compared with delivering the same dose from an unmodulated field. It therefore follows that the total body dose due to leakage radiation will be increased^1,12,13^. It is estimated that an additional 0.25% of surviving patients will develop a radiation-induced malignancy because of this factor^1^. Furthermore, the IMRT dark current is approximately sevenfold higher than that of conventional radiotherapy, leading to further increases in the exposure of normal tissue to low-dose irradiation that might induce lethal damage^14–16^. Followill and colleagues at the M. D. Anderson Hospital made estimates of whole-body dose equivalent resulting from IMRT^17^. They concluded that, compared with conventional radiotherapy, IMRT may approximately double the risk of secondary cancers from 0.4 to 1%. In our study, the patients in IMRT were treated with five to seven radiation fields, while there were two in the RT group. Although the difference in the accumulated radiation doses between the two groups was not statistically significant (*P* = 0.068), the doses of normal tissue or surrounding OAR were lower in IMRT group and the exposure volumes of normal tissue were bigger. The leakage dose and dark current were also increased in the IMRT group.

In our study, the mean latency between IMRT and development of SPSCC was 4.20 years, which was shorter than RT group (7.65 years), and receiving IMRT was related positively to an increased risk of SPSCC (HR 4.25; 95% CI 1.92–9.40; *P* < 0.001). We found that more than half (50.33%) of SPSCC occurred within 3 years in IMRT group, whereas 56.52% patients developed SPSCC after a latency of more than 10 years in RT group. It indicated that a careful and adequately follow-up protocol, especially in the first three years, should be designed for NPC patients with IMRT and the radiation-exposed region should be examined.

In this study, we noted that the most common tumor site in the SPSCC was the oral cavity, accounting for approximately 80% of cases both in IMRT group (12/15) and RT group (19/23). Interestingly, we observed a distinctly different pattern of tumor location. Among the oral cavity SPSCC identified, 75% (6/12) were found at gingiva and bucca cavioris in IMRT group, whereas 73.68% (14/19) were found at tongue and hard palate in RT group. Such a pattern change may be attributable to the difference in oral cavity dose distribution between the two radiotherapy techniques. However, some of the dose-volume parameters of the oral cavity in the RT group in 1970–1990 were missing in our study. Theoretically, IMRT has advantages over conventional radiotherapy with respect to reducing both the high-dose region of the tongue and the risk of oral mucositis. However, with the use of multiple beam arrangements and the consistent inclusion of level Ib nodal group as treatment targets, IMRT produces plans with wider spread of low-dose volumes covering the anterior and lateral edges of oral cavity^18^. This change in locations of SPSCC along with the transition of radiotherapy techniques carries potential clinical implications.

In our study, the univariate analysis revealed that there was no significant correlation between receiving IMRT and the survival of SPSCC (*P* = 0.051). Treatment modalities was the independent prognostic factors affecting survival of SPSCC in NPC patients^5^. Our study is subject to the limitations inherent in all retrospective studies, including a relatively small number of patients who were treated with various regimens over time. Therefore, prospective studies with larger samples need to be performed.

## Conclusions

Receiving IMRT was related positively to an increased risk of SPSCC. The latency was shorter than patients who had received RT. It indicated that a careful and adequately follow-up protocol, especially in the first three years, should be designed for NPC patients with IMRT and the radiation-exposed region should be examined. There was no significant correlation between receiving IMRT and the survival of SPSCC.

## Data Availability

The datasets used and analysed during the current study available from the corresponding author on reasonable request.
