# Author Correction: Clinicopathologic characteristics of second primary squamous cell carcinoma in patients with nasopharyngeal carcinoma after intensity-modulated radiotherapy

**DOI:** 10.1038/s41598-023-36809-7

**Published:** 2023-06-15

**Authors:** Xi Wang, Shunlan Wang, Yang Cao, Chunqiao Li, Caishan Fang, Weiping He, Zhuming Guo

**Affiliations:** 1 Department of Otolaryngology, Guangzhou University of Traditional Chinese Medicine First Affiliated Hospital, Guangzhou, China; 2 The First School of Clinical Medicine, Guangzhou University of Chinese Medicine, Guangzhou, China; 3 Department of Oncology, Guangzhou University of Traditional Chinese Medicine First Affiliated Hospital, Guangzhou, China; 4 Department of Head and Neck, Sun Yat-Sen University Cancer Center, Guangzhou, China

Correction to: *Scientific Reports* https://doi.org/10.1038/s41598-023-34848-8, published online 20 May 2023

The original version of this Article omitted an affiliation for Xi Wang. The correct affiliations are listed below.

Department of Otolaryngology, Guangzhou University of Traditional Chinese Medicine First Affiliated Hospital, Guangzhou, China.

The First School of Clinical Medicine, Guangzhou University of Chinese Medicine, Guangzhou, China

The original Article has been corrected.

